# *VraSR* Regulatory System Contributes to the Virulence of Community-Associated Methicillin-Resistant *Staphylococcus aureus* (CA-MRSA) in a 3D-Skin Model and Skin Infection of Humanized Mouse Model

**DOI:** 10.3390/biomedicines10010035

**Published:** 2021-12-24

**Authors:** Nilakshi Barua, Ying Yang, Lin Huang, Margaret Ip

**Affiliations:** 1 Department of Microbiology, Faculty of Medicine, The Chinese University of Hong Kong, Prince of Wales Hospital, Sha Tin, N.T., Hong Kong 999077, China; nilakshibarua@cuhk.edu.hk (N.B.); claire_yang@link.cuhk.edu.hk (Y.Y.); 2 Division of Plastic, Reconstructive and Aesthetic Surgery, Department of Surgery, The Chinese University of Hong Kong, Prince of Wales Hospital, Sha Tin, N.T., Hong Kong 999077, China; huanglin@surgery.cuhk.edu.hk

**Keywords:** *VraSR* regulatory system, 3D Skin model, humanized mouse model, *Staphylococcus aureus*, MRSA, skin and soft-tissue infections

## Abstract

The vancomycin-resistance associated sensor/regulator, *VraSR* two-component regulatory-system (*VraSR*), regulates virulence and the response of *Staphylococcus aureus* (SA) to environmental stress. To investigate the role of *VraSR* in SA skin and soft tissue infections (SSTI), we inactivated the *VraSR* of a clinical CA-MRSA ST30 strain by insertional mutation in *vraR* gene using the TargeTron-Gene Knockout System. We constructed an organotypic keratinocyte fibroblast co-culture (3D-skin model) and a humanized mouse as SSTI infection models. In the 3D-skin model, inactivation of *VraSR* in the strains ST30 and USA300 showed 1-log reduction in adhesion and internalization (*p <* 0.001) compared to the respective wildtype. The mutant strains of ST30 (*p <* 0.05) and USA300-LAC (*p <* 0.001) also exhibited reduced apoptosis. The wildtype ST30 infection in the humanized mouse model demonstrated increased skin lesion size and bacterial burden compared to BALB/c mice (*p <* 0.01). The response of the humanized mouse towards the MRSA infection exhibited human similarity indicating that the humanized mouse SSTI model is more suitable for evaluating the role of virulence determinants. Inactivation of *VraSR* in ST30 strain resulted in decreased skin lesion size in the humanized mouse SSTI model (*p <* 0.05) and reduction in apoptotic index (*p <* 0.01) when compared with the wildtype. Our results reveal that inactivating the *VraSR* system may be a potent anti-virulence approach to control MRSA infection.

## 1. Introduction

Methicillin-resistant *Staphylococcus aureus* (MRSA) is a major bacterial pathogen causing hospital infections, as well as community infections worldwide [1]. The emergence of community-associated (CA-MRSA) has increased the burden of staphylococcal disease worldwide. Ninety percent of cases of CA-MRSA infections are skin and soft-tissue infections [2]. The MRSA, ST30, is the predominant clonal type isolated among patients with skin and soft tissue infections (SSTI) in Hong Kong [3] compared to MRSA USA300 that is prevalent in the United States [4].

During the establishment of an infection, *S. aureus* (SA) expresses a wide range of virulence factors regulated by the Two-component regulatory systems (TCRSs) and Sar family [5]. Among the diverse TCRSs present in SA, the *Vra*S and *Vra*R proteins enable the bacteria to respond to and survive environmental stress. *Vra*S and *Vra*R TCR is required for resistance to cell wall antibiotics, e.g., methicillin [6]. The *Vra*T (previously YvqF) is required by *Vra*S and *Vra*R to induce a global transcriptional response to cell wall stress and cell wall acting antibiotics [7]. 

The *VraSR* regulatory system directly regulates the accessory gene regulator (*agr)* operon, a major virulence regulator in SA, which regulates many virulence factors, including α-Hemolysin (Hla) and phenol-soluble modulins (PSMs), thus suggesting that *VraSR* regulatory system possesses a wide range of regulatory function [8]. Some of the toxins of SA are found to be host specific, such as the bi-component toxins Panton Valentine leukocidin (PVL) and Bi-component gamma-hemolysin (HlgCB) [9]. PVL are more compatible with human type and rabbit type receptors, but HlgCB shows high affinity towards human type receptor only [10]. Except bi-component toxins, the super-antigens and surface proteins are more compatible with human cells compared with mouse cells [11]. Therefore, development of a model that can translate the findings from mice to humans is required. This could be achieved via a humanized mouse model that mimics the human immune system and is more suitable to study staphylococcal virulence, therapy, and vaccine efficacy [10]. In cell culture systems, the bi-dimensional nature of keratinocyte monolayer culture lacks the complex stratification, terminal differentiation of epidermal tissue, and fails to provide a comprehensive insight into the colonization and invasion by SA. In the present study, we aim to gain insight into the role of *VraSR* system in SA skin and soft tissue infections. We inactivated the *VraSR* system in a clinical strain of CA-MRSA representing the serotype ST30 by constructing a *vraR* mutant via insertional mutagenesis using the Targetron gene knockout system. We constructed an organotypic keratinocyte fibroblast co-culture model (3D-skin model) and a humanized mouse model to study the effect of the inactivation of *VraSR* regulatory system on the virulence of the SA.

## 2. Materials and Methods

### 2.1. Bacterial Culture and Reagents

The bacterial strains used in this study were a clinical CA-MRSA (Southwest Pacific clone, ST30, spa t019) strain, USA300 LAC (NARSA wildtype, ST8, spa t008), and its isogenic mutant defective in *vra*R. The strains were grown at 37 °C with shaking in BHI (Oxoid Hampshire, UK) or tryptic soy (TS, Oxoid, Hampshire, UK) broth, or blood agar. Optical density at 600 nm (OD_600nm_) was taken to prepare the bacterial inocula and was verified by plating on blood agar plates to determine CFUs. Unless otherwise stated, all the other reagents were obtained from Sigma(St. Louis, MO, USA).

### 2.2. Construction of Isogenic Knockout Mutant Strain

The *vra*R knockout of ST30 CA-MRSA was constructed using the TargeTron Gene Knockout System (Sigma) by insertional mutations [12]. Briefly, overlap extension PCR was performed using JumpStart REDTaq ReadyMix (Sigma, St. Louis, MO, USA) with IBS primer, EBS1d, EBS2 primer (Supplemental Table S1) and intron PCR template. The PCR reaction cycle 94 °C 30 s, 30 cycles of 94 °C 15 s, 55 °C 30 s, 72 °C 30 s, and 72 °C 2 min was used. The PCR products were purified using the iNtRON PCR Product Purification Kit (Tech Dragon, Hong Kong). The purified PCR products (1 μg) and pNL9164 plasmid (1 μg) were digested with HindIII and BsrGI. The purified digested products were ligated with T4 DNA ligase (NEB) and transformed to the *E. coli* competent cell SA30B (Lucigen, Middleton, WI, USA) by heat shock. Competent ST30 CA-MRSA cells were prepared as described by Monk et al., 2012 [13] and the recombinant plasmid was electroporated at room temperature with pulse at 21 kV/cm, 100 Ω, and 25 μF using Bio-Rad Gene Pulser II Electroporator (Hercules, CA, USA). The recombinants were then cultured in blood agar containing 10 μg/mL erythromycin overnight at 37 °C. Intron insertion was confirmed by PCR with flank F and flank R primers (Supplemental Table S1). The pNL9164 plasmid was cured by incubating the plate at 43 °C. The isogenic mutant strain was confirmed through PCR of the target gene and the size band of the intron insertion and by sequencing. 

### 2.3. Generation of a 3D-Skin Model

A 3D-skin model was constructed in Corning Transwell polyester membrane 12 mm inserts with 0.4 µm pores according to our previously described method [14]. The human keratinocytes and fibroblasts were obtained from discarded surgical foreskins from a single donor and stored in liquid nitrogen until use. Ethical approval was obtained from the respective Institutional Research Ethics Committee (CRE-2004.433, 31052004 and CRE-2006.434, 26062006). Collagen gel was prepared using rat-tail type-I collagen (ibidi, Gräfelfing, Munich) to a final concentration of 3 mg/mL in 1.2× DMEM neutralized with 5 M NaOH. The fibroblast cells were mixed with the collagen gel to obtain a final cell density of 1 × 10^5^ cells/mL to generate the fibroblast-populated collagen lattice (FPCL) and 600 μL of this mixture was loaded into transwells. The FPCL was polymerized at 37 °C, 5% CO_2_ for 2 h and equilibrated in keratinocyte growth medium (KGM) overnight (composition in Supplementary Section S1). After 24 h equilibration, 200 µL KGM containing 2 × 10^5^ keratinocytes were seeded on top of the FPCL. After 24 h of seeding the insert was lifted to an air–liquid interface and cultured for an additional 1 week with KGM being changed every 48 h. All experiments were performed in triplicates on 1-week air-exposed cultures. 

### 2.4. Evaluation of MRSA Infection in 3D-Skin Model 

The 3D-skin model was infected by adding bacterial suspension containing 2 × 10^7^ CFU in 100 µL of KGM media on top of the skin model and incubated for 1 h at 37 °C. Non-adherent/loosely adherent bacteria were removed and fresh media were added. Media from the culture were then collected at 2 h, 24 h and 48 h of infection. To enumerate adherent and internalized bacteria, the skin model was cut in two equal halves. One-half was homogenized in PBS using a glass Potter–Elvehjem tissue homogenizer(Sigma, Allentown, PA, USA). Bacterial number was assessed by serial dilution and plating of the bacteria for CFU counts. The other half of each skin model was fixed in 4% (*v*/*v*) formaldehyde, dehydrated, and then embedded in paraffin. The paraffin blocks were cut into 5 μm sections followed by deparaffinization and rehydration for haematoxylin and eosin (H and E) staining, and terminal deoxynucleotidyl transferase dUTP nick end labeling (TUNEL) assay.

TUNEL assay was performed following the manufacturer’s instructions of Click-iT Plus TUNEL assay for in situ apoptosis detection with Alexa Fluor dyes (Life Technologies, Burlington, ONT, Canada). Anti-*Staphylococcus aureus* antibody (Abcam, Cambridge, UK) and Goat Anti-Rabbit IgG H&L conjugated with Alexa Fluor 568 (Abcam, Cambridge, UK) secondary antibody was used to study the dissemination of the bacteria into different strata of the skin model. DNA stain Hoechst (Sigma, St. Louis, MO, USA) was used as a counter stain. The whole area of the sections was scanned for apoptotic keratinocytes. The apoptotic index was then calculated using the following formula

(1) $$\mathrm{AI}\;=\frac{\mathrm{Number}\;\mathrm{of}\;\mathrm{apoptotic}\;\mathrm{keratinocytes}\;\mathrm{per}\;\mathrm{section}}{\mathrm{Total}\;\mathrm{number}\;\mathrm{of}\;\mathrm{keratinocytes}\;\mathrm{per}\;\mathrm{section}}\times 100$$

### 2.5. Determination of Cytokine Levels 

The levels of interleukin-1 (IL-1α), IL-1β, IL-6, and tumor necrosis factor alpha (TNF-α) in the culture medium at each time point was determined by ELISA (Biolegend, San Diego, CA, USA) following manufacturer’s protocol. 

### 2.6. Generation of Humanized Mice 

Animal experiments were performed with permission of the Animal Experimentation Ethics Committee (AEEC) of The Chinese University of Hong Kong. The study was approved by the University Animal Experimentation Ethics Committee (AEEC; Reference no.: 16-158-MIS; 06062016) and conducted at The Laboratory Animal Services Centre in compliance with the International Guiding Principles for Biomedical Research Involving Animals and The Hong Kong Code of Practice for Care and Use of Animals for Experimental Purposes. The approval of clinical ethics for the umbilical cord blood was obtained from the Joint Chinese University of Hong Kong-New Territories East Cluster Clinical Research Ethics Committee (CRE-2018.261). 

The humanized mouse was generated as described previously [15]. Mononuclear cells (MNCs) were enriched from Human umbilical cord blood (hUCB) using Ficoll–Paque PREMIUM density gradient centrifugation (GE Healthcare, Chicago, IL, USA). MNCs were enriched for hCD34+ cells using a human CD34 MicroBeadKit UltraPure and the purity of hCD34+ cells was determined using a FACSCanto-II flow cytometer.

Four-week-old NOD/SCID mice and 12-week-old BALB/c mice were obtained from the Laboratory Animal Services Centre (LASEC) of The Chinese University of Hong Kong and housed in individual ventilated cages (IVC) under the conditions of 22–25 °C and a 12 h light-dark cycle, with free access to chow and water. NOD/SCID mice were used for generation of humanized mice model and busulfan at 30 mg/kg was administered twice every 24 h by intraperitoneal injection for myelosuppression. Human CD34+ hematopoietic stem cells (HSC) were resuspended in cold PBS at 4 °C at a concentration of 5 × 10^5^ CD34+ cells in 100 µL and injected to lateral tail vein of adult mice. 

Engraftment of HSC was assessed after 14 weeks. Peripheral blood drawn from mice was stained with anti-human CD45, anti-mice CD45 (BD Pharmingen, San Diego, CA, USA) and analyzed by flow cytometry (BD FACSAria, Franklin Lakes, NJ, USA). The data was analyzed using BDTM cytometer. Human engraftment levels are defined as the proportion of total nucleated cells that stain positive for human CD45 and level of engraftment equal to % CD45+ human cells divided by the sum of % CD45+ human cells plus % CD45+ mouse cells. Levels of human CD45+ cells reaching 25% in the blood were considered as successfully engrafted mice (Supplementary Figure S1) [16]. 

### 2.7. Cytotoxicity Assay 

Bacterial supernatant of SA (10% final volume) was added to 96 well tissue culture plate containing 10^5^ neutrophils and incubated at 37 °C for 3 h. Cytotoxicity was determined by alamar Blue (Life Technologies, Burlington, ONT, Canada). Fluorescence emission was measured at 595 nm with excitation at 535 nm.

RBCs and neutrophils were isolated from the whole blood of healthy individuals (obtained from the Hong Kong Red Cross Blood Transfusion Service) and rabbit (obtained from the Laboratory Animal Services Centre of The Chinese University of Hong Kong), humanized mice, BALB/c mice. For neutrophil-isolation, density gradient centrifugation using Ficoll–Paque Plus (GE Health, Chicago, IL, USA) and dextran sedimentation was used according to the manufacturer’s instruction. The RBCs were transferred to a new tube and washed with PBS at 3000× *g* for 2 min, until the supernatant was clear. The neutrophils were resuspended in 10 mL RPMI 1640 with 10% FBS at the density of 1 × 10^6^ cells/mL. One hundred microlitres bacterial supernatant of SA were added to 1.5 mL tubes containing 20 µL RBCs and 880 µL PBS. Autoclaved dH_2_0 was used as positive control. Tubes were incubated at 37 °C for 30 min, followed by at 3000× *g* for 2 min. The optical density of supernatant was measured at 595 nm. 

### 2.8. Humanized Mouse MRSA-SSTI Model

The virulence of wildtype MRSA and respective isogenic strains were tested in humanized mice and BALB/c mice. Overnight cultures of mutant and the parental strain were diluted in fresh tryptic soy broth (TSB) and incubated at 37 °C with shaking at 200 rpm till mid-log phase. Then, the cells were harvested by centrifugation at 3600 rpm for 10 min, washed, and resuspended in PBS to a concentration of 1 × 10^8^ CFU/mL. For the SSTI model, 100 μL of the suspension was subcutaneously injected into shaved flank. 

Skin-lesions were defined by darkened areas of necrosis. The lesion size was quantitated using the ruler technique (RT). The length (longest head-to-toe length) and the width (largest width perpendicular to the length) of the lesions were measured manually. The size was calculated by multiplying the length and width of the lesion. The skin tissues were excised and homogenized in 1 mL PBS. The homogenate was centrifuged at 12,000× *g* for 10 min and CFU was determined on blood agar plates.

### 2.9. Confocal Laser Scanning Microscopy (CLSM) Analysis of Biofilms

Single colony of the respective overnight bacterial cultures were used to inoculate 5 mL of brain heart infusion (BHI) media and incubated for 16 h. The bacterial cultures were then diluted to 1:200 in BHI supplemented with glucose (1% *w*/*v*) and 500 µL were added to each well of the Nunc LabTek II 8 well chamber and incubated statically at 37 °C for 24, 72, and 120 h in a humidified chamber. At each time point, the chambers were washed three times with PBS followed by fixation of biofilm with 4% formalin for 15 min. After the formalin fixation, biofilms were washed and stained with the LIVE/DEAD BacLight Bacterial Viability Kit (Invitrogen, Waltham, MA, USA), following the manufacturer’s instructions. Slides were then mounted using ProLong Diamond Antifade Mountant (Invitrogen). Micrographs were acquired with a confocal laser-scanning microscope (CarlZeiss LSM880 Laser Confocal Microscope, Oberkochen, Germany) by sequentially scanning with a 488 nm Argon laser for excitation. The emitted fluorescence of Syto9 was recorded within the 505–530 nm range. The Z-stacks were captured every 10 μm section from the bottom of the biofilm at different areas in the well. The Carl Zeiss Zen3.2 (blue edition, Oberkochen, Germany) was used to analyze the images [17].

## 3. Results

### 3.1. Construction of Isogenic Knockout Mutant Strain ST30ΔvraR

The isogenic knockout mutant strain of ST30 was established by insertional mutation using the TargeTron Gene Knockout System (Sigma, St. Louis, MO, USA). The insertion of the intron was carried out at the position between the nucleotides 237 and the 238 of the *vra*R gene in an antisense orientation. After a knockout was confirmed, the pNL9164 plasmid was cured at 43 °C overnight since it carried a temperature-sensitive origin of replication pT181 cop-634 ts repC4. A PCR using pNL9164 seq F and pNl9164 seq R primers was carried out to check for the absence of the plasmid (Supplementary Figure S2).

### 3.2. Infection of the Skin Model 

**Figure 1:**
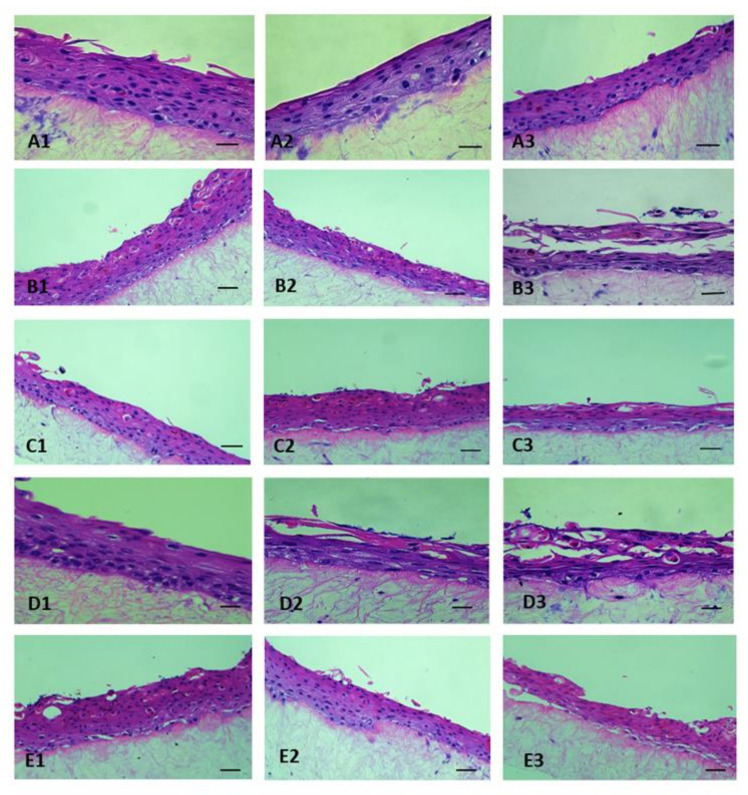
D-skin models at (**1**) 2 h, (**2**) 24 h, and (**3**) 48 h after inoculation with (**A**) PBS (**B**) ST30, (**C**) ST30Δ*vraR*, (**D**) USA300, and (**E**) USA300Δ*vraR.* Scale bar shows 50 µm.

After 1-week of air-liquid interface culture the 3D-skin model was generated. The differentiation of the keratinocytes to strata basale, strata spinosum and strata corneum was observed by H and E staining. Wildtype ST30, USA300 and their respective isogenic mutant ST30Δ*vraR* and *USA*300Δ*vraR* were used to infect the 3D-skin model. Exfoliation in the skin model was observed at 24 h and 48 h by wildtype ST30 and USA300. The isogenic mutants ST30Δ*vraR* and USA300Δ*vraR* colonized the 3D-skin model as depicted by the microcolonies but did not induce tissue damage by exfoliation (Figure 1).

**Figure 2:**
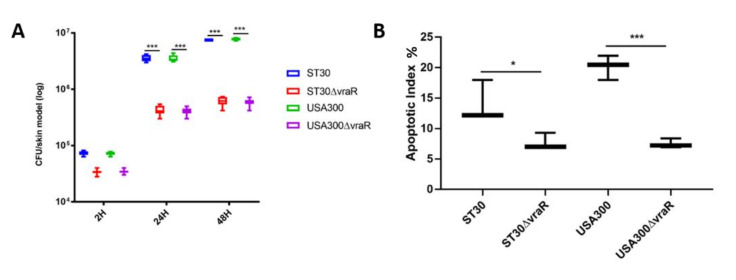
(**A**) The bacterial enumeration of adherent and internalized bacteria upon infection of the 3D-skin model. Data are shown as the means ± SD, significance were determinant by two-way ANOVA followed by Dunnett’s multiple comparison test. The *p*-values were obtained by comparing the mean of the treatments of the skin model at each time point and coded as *** p < 0.001. (**B**) The apoptotic index was measured to determine the amount of cell death induced by the bacterial infection. The error bars represent the standard deviation of the mean values. Significance was obtained by one-way ANOVA followed by Dunnett’s multiple comparison test. The *p*-values were obtained by comparison between the mean of the treatments of the skin model and coded as * p < 0.05 and *** p < 0.001.

Bacteria were enumerated at 2 h, 24 h, and 48 h to study the adherence and internalization of the bacteria (Figure 2A). At 2 h after infection, no significant difference was observed in bacterial internalization for the wildtype ST30 and USA300 strain. At both 24 h and 48 h after infection, the internalization of the isogenic mutant ST30Δ*vraR* was significantly lower than its wildtype ST30 strain (*p <* 0.001). At 24 h and 48 h, the isogenic USA300 Δ*vraR* mutant internalization was lower than its wildtype USA300 (*p <* 0.001). 

**Figure 3:**
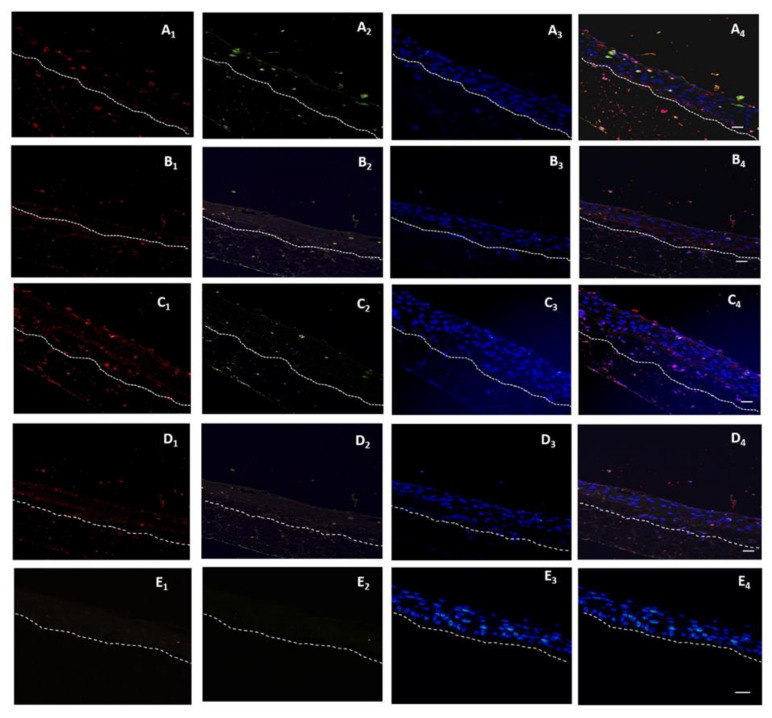
A double-labeling assay for detection of the staphylococcal invasion of the skin model with anti-*Staphylococcus aureus* antibody and detection of the apoptotic cells, using TUNEL assay after 48 h of infection the MRSA. (**A**) ST30 and (**B**) ST30Δ*vraR*, (**C**) USA300 and (**D**) USA300Δ*vraR*, (**E**) PBS. *(***1**) the anti-*Staphylococcus aureus* antibody with goat anti-rabbit IgG H and L conjugated with Alexa Fluor 568 secondary antibody, (**2**) the Click-iT^®^ TUNEL Alexa Fluor^®^ 488 cells (**3**) Hoecsht stain, and (**4**) the overlay of the emission signals. White hashed line demarcates the dermal epidermal boundary between the strata basale and the collagen gel populated with fibroblasts. Scale bar shows 50 µm.

The modulation of the cell death in the 3D-skin model was analyzed by TUNEL stain (Figure 3). Dissemination of the bacteria in the case of all the four bacterial strains was observed across three strata of the 3D-skin model. Cell death induced by the wildtype strains ST30 (*p <* 0.01) and USA300 (*p <* 0.001) as illustrated by apoptotic index, was significantly higher in comparison to their respective isogenic Δ*vraR* isogenic mutants (Figure 2B). 

### 3.3. Cytokines

**Figure 4:**
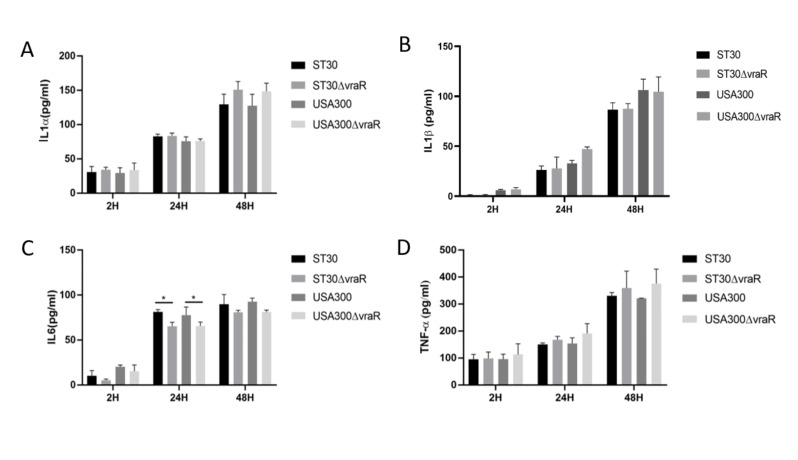
Cytokines IL1α (**A**), IL1β (**B**), IL6 (**C**), and TNFα (**D**) released by the skin model were measured at 2 h, 24 h, and 48 h, and normalized to the values measured for the uninfected skin model. The error bars represent the standard deviation of the mean values. Significance was determined by two-way ANOVA. Dunnett’s multiple comparison test was performed to analyze the release of cytokines by the skin model in response to each respective treatment. The *p*-values were obtained by comparison between the mean of the treatments of the skin model at each time point and coded as * p < 0.05.

Cytokines, IL-1α, IL-1β, and TNFα released into the medium of the 3D-skin model among the tested groups did not vary at 2 h (Figure 4). At 24 h the release of IL-6 significantly differed in the wildtype and mutant in both ST30 (*p <* 0.05) and USA300 (*p <* 0.05). The release of TNFα did not significantly vary between the mutants and the respective wildtype strain.

### 3.4. Humanized Mouse MRSA-SSTI Model

**Figure 5:**
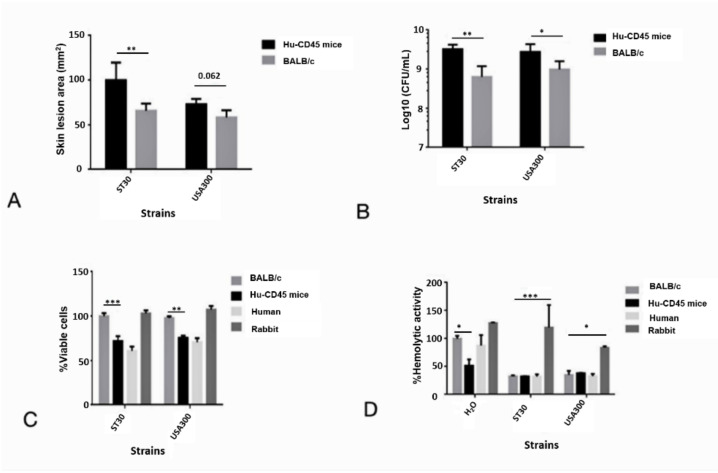
Humanized mice were more susceptible to MRSA ST30 strain. (**A**) The skin lesion sizes were defined by darkened areas of necrosis and calculated by V = Πx (L × W)/2, V is the size of the lesion, L is the length of the lesion, and W is the width of the lesion. Wildtype USA300 was used as control. Hu is the humanized mice. (**B**) Bacteria burden of mice after infection. (**C**) Neutrophil lysis activity of culture filtrate. Neutrophil lysis activity of wildtype ST30 strain in BALB/c was normalized to 100%. The error bars represent the standard deviation of the mean values, significance was determinant by two-way ANOVA (**D**) Hemolysis by bacterial culture filtrate. Hemolytic activity of H_2_O in BALB/c was normalized to 100%. The error bars represent the standard deviation of the mean values, significance was determinant by Two-way ANOVA. * indicate the significant difference between humanized mice and rabbit within each strain, and coded as * *p* < 0.05, ** *p* < 0.01, *** *p* < 0.001.

All infected humanized mice and BALB/c mice exhibited visible skin lesions. Humanized mice were more susceptible towards SA infection with increased lesion size and bacterial burden than BALB/c mice (Figure 5A,B). As polymorphonuclear (PMN) cells were reported to play key role in immunity against SA acute infection, the PMN and red blood cells (RBC) from different hosts were isolated and incubated with supernatant from the early stationary culture of SA. The PMN and RBC of the humanized mouse responded to MRSA supernatant similarly like that of the human PMN and RBC. Cytotoxicity assay revealed that PMN preparations from humans and humanized mice exhibited significant sensitivity to MRSA culture filtrate than BALB/c mouse (Figure 5C). However, no significant difference was found in haemolytic activity of the MRSA culture filtrate on the RBC derived from humanized mouse and BALB/c mouse, rabbit RBC still being the most fragile compared to the RBCs derived from human, humanized mice and BALB/c mice respectively (Figure 5D). 

**Figure 6:**
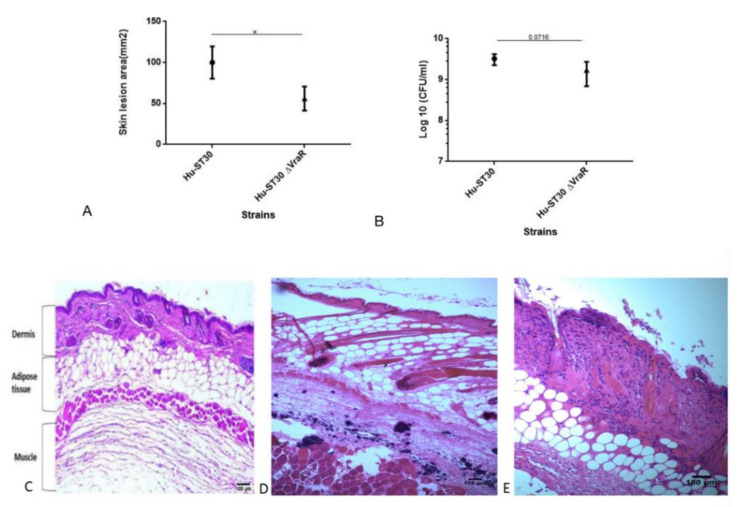
Deletion of *VraR* in MRSA ST30 strain showed reduced bacterial virulence. (**A**) Mice infected with wildtype ST30 or ST30Δ*vraR*. The skin lesion sizes were defined by darkened areas of necrosis and calculated by V = Πx (L × W)/2, V is the size of the lesion, L is the length of the lesion, and W is the width of the lesion. Hu depicts humanized mice. (**B**) Bacteria burden of mice after infection. The error bars represent the standard deviation of the mean values; significance was determined by one-way ANOVA, and coded as * *p* < 0.05. (**C**,**D**) Haematoxylin and eosin staining of the MRSA skin and soft-tissue infection mouse model. Mice were inoculated subcutaneously with either PBS or MRSA as described in Methods. (**C**) PBS control, (**D**) ST30 infected, and (**E**) ST30△*vraR* infected. Original magnifications of images are 100×.

The ST30 induced skin lesion size in the inoculated humanized mouse was significantly increased when compared with its isogenic ST30ΔvraR mutant strain (*p* < 0.05) (Figure 6A). No significant difference in CFU between the humanized mouse infected with wildtype ST30 and ST30ΔvraR mutant strain, suggesting that the reduced skin lesion size in the mutant is not due to a reduced bacterial burden (Figure 6B). No significant difference in the release of cytokines was observed upon infection with wildtype ST30 and ST30ΔvraR (Supplementary Figure S3). From the results above, we found that inactivation of VraSR reduced the MRSA virulence.

**Figure 7:**
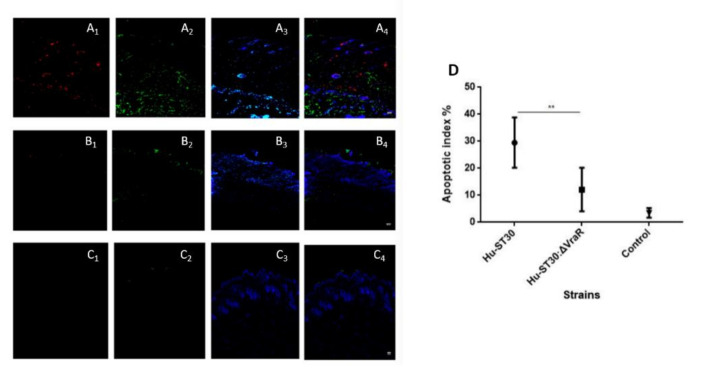
Comparison of MRSA ST30 and ST30ΔvraR using skin and soft-tissue infection (SSTI) model. Mice were inoculated subcutaneously with either PBS or bacteria as described in Methods. (**A**) A double-labeling assay for detection of the staphylococcal invasion of the skin model with anti-*Staphylococcus aureus* antibody and detection of the apoptotic cells, using TUNEL assay after 48 h of infection with the MRSA. (**A**) ST30 and (**B**) ST30Δ*vraR*, and (**C**) PBS (**1**) anti-*Staphylococcus aureus* antibody with goat anti-rabbit IgG H and L conjugated with Alexa Fluor 568 secondary antibody, (**2**) the Click-iT^®^ TUNEL Alexa Fluor^®^ 488 cells (**3**) Hoecsht stain, and (**4**) the overlay of the emission signals. White hashed line demarcates the dermal epidermal boundary between the strata basale and the collagen gel populated with fibroblasts. Scale bar shows 50 µm. (**D**) Apoptotic index. Ten fields were calculated for each condition and original magnifications of images are 100×. Data are shown as the means ± SD, significance were determinant by one-way ANOVA and coded as ** *p* < 0.01.

To evaluate whether inactivation of *VraSR* decreased cell necrosis or apoptosis, H and E staining (Figure 6C,D) and TUNEL assay (Figure 7A–C) of the skin lesion was evaluated. H and E staining of the SSTI mouse model showed necrotic epidermis and abscesses. ST30 infection showed damage to the dermis and fat tissue. Bacterial colonies formed in the muscle (Figure 6D). The *VraSR* inactivation reduced dermonecrosis. No damage to the structure of dermis and fat tissue was observed when compared to the wildtype ST30 infected group (Figure 6E). 

TUNEL assay together with Anti-*S. aureus* antibody showed the bacteria (red) and the apoptosis (green) were spread across dermis to muscle (Figure 7A). For the ST30Δ*vraR* (Figure 7B) mutant strain infection, the apoptosis existed only in the dermis and the amount of bacterial colony was reduced than the wildtype infected group. The apoptotic index (AI) was decreased in the ST30Δ*vraR* mutant group, when compared with the wildtype group, and indicated the role of *VraSR* in the virulence of ST30 strain (Figure 7D). 

### 3.5. CSLM Analysis of Biofilm Formation

**Figure 8:**
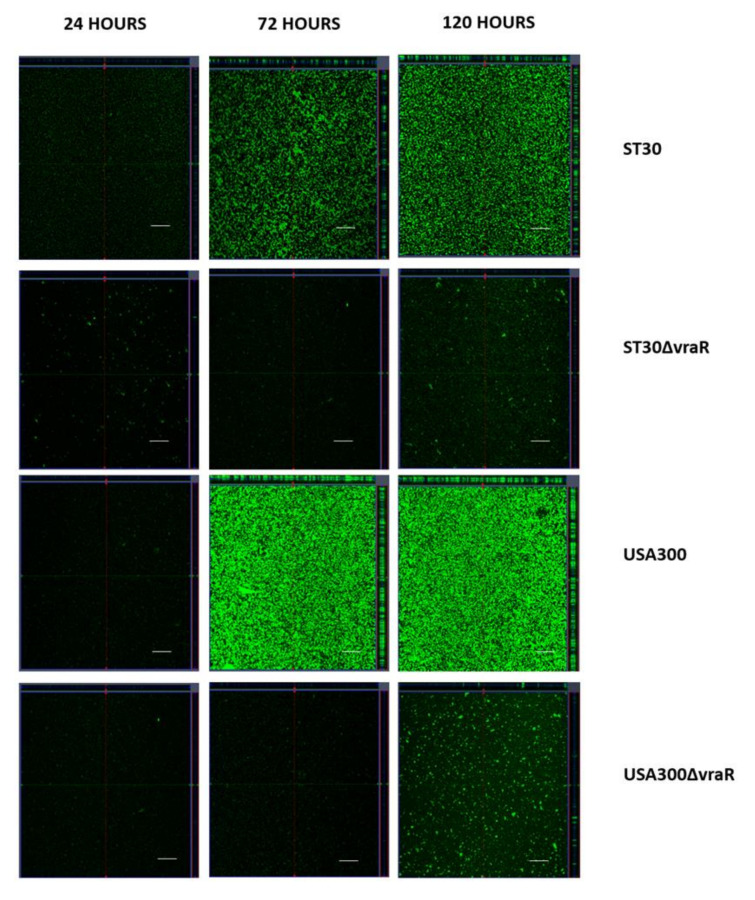
USA300 and their isogenic mutants ST30ΔvraR and USA300ΔvraR. The central panels represent the x–y plane, and the top and right side panels represent the x–z and y–z planes, respectively. Scale bars represent 50 μm.

The biofilms were stained with Syto9 at 24 h, 72 h, and 120 h and were analyzed by CSLM. Figure 8 shows the acquired confocal images of ST30, USA300, ST30Δ*vraR*, and USA300Δ*vraR*. The wildtype strains ST30 and USA300 showed a confluent growth and increased biovolume (xy-plane) at 72 and 120 h in comparison to their respective isogenic mutants. The expansion of the biofilm of the wildtype during the period from 24 h to 120 h was multidirectional, in both xy-plane (increase in surface coverage) and z projection (increase in thickness). At 72 h the biofilms formed by the wildtype ST30 and USA300 covered most of the surface available. Isogenic mutants revealed microcolonies scattered on the surface at both 72 and 120 h. The z-projection of the xy-stacks revealed difference in thickness of the biofilm between the wildtype and their isogenic mutants. The wildtype produced compact biofilm with enhanced thickness, which was not observed in their respective isogenic mutants.

## 4. Discussion

CA-MRSA is a major cause of skin and soft tissue infection [18]. TCRSs, which include the GraRS [19], SaeRS [20], *VraSR* [21], and ArlS–ArlR [22] regulate the response of SA to the external stimulus. Hence, investigation of the direct and non-direct regulation virulence by TCRSs in SA is essential. Recent studies revealed that bacteria utilize TCRSs to promote survival during infection via evasion of the host immune response. These include *VraSR* TCRS system in SA*, Streptococcus suis* and PhoPQ TCRS in *Yersinia pestis* [23,24]. In MRSA, *VraSR* is highly expressed in VISA/hVISA strains and play a vital role in immune evasion of SA by defending against phagocytosis [25]. However, the role of *VraSR* in skin and soft tissue infection is not clear. Our study provides evidence that *VraSR* plays a vital role in regulating adhesion and establishing skin and soft tissue infection. 

We established a 3D-skin model to study the interactions during infection by MRSA ST30, USA300 and their respective isogenic mutants Δ*vraR.* The model enabled detailed examination of SA invasions at various strata during the course of time of infection and minimized alterations with exogenous replenishment of media and rapid cell death of monolayers as compared to primary keratinocytes alone. 

Previous studies revealed the downregulation of the adhesion-associated genes, fibrinogen-binding protein *A (fnbB),* fibrinogen-binding protein *(fnbA),* clumping factor A *(clfA),* and elastin binding protein *(ebps))* in Δ*VraSR* of SA [26], and *Streptococcus suis* [23] indicating that *VraSR* plays a role in adherence to host cells. In our current study, we also observed that the inactivation of the *VraSR* in ST30 and USA300 reduced the ability to adhere, induced damage to the 3D-skin model, and reduced the ability to establish skin infection in the humanized mouse model. Consistent with previous studies, our findings indicate that *VraSR* is involved in expression virulence factors required to establish SSTI.

*VraSR* was also reported to be associated with bacterial biofilm formation, and inactivation of *VraSR* showed reduced adhesion and decreased biofilm forming ability. *Agr* operon is involved in the regulation of biofilm formation and virulence in SA*. VraSR* binds to the promoter region of *agr* operon thus regulating the virulence of SA [26]. By CSLM analysis of the biofilm formation by the wildtype and their respective mutant strains, we also observed that inactivation of the *VraSR* impaired the biofilm formation. 

The use of mouse model has provided a platform for investigating the virulence of SA and the identification of molecules that have potential as human vaccine candidates. However, findings in mice may not translate to humans due to differences between the murine and human immune system [27,28]. Failures of human clinical trials suggest that the mouse is not an appropriate model for studying SA infections in humans. A humanized mouse model generated with non-obese diabetic (NOD)/severe combined immune deficiency (SCID)/IL2rγnull (NSG) mice has now become one of the favored models for host–pathogen interactions, as these mice accept human hematopoietic cells (CD34+) and s mimic human immune system [29]. Humanized mouse models have been successfully applied to study various infections, such as HIV, *Salmonella*, *Shigella*, and *Mycobacterium,* playing an important role in preclinical translational research [30]. Humanized mouse models have been proposed to study SA infection to explore human-specific virulence factors of SA as well as the components of the human immune system in protection against SA infection. 

Our study demonstrates that humanized mice are more sensitive to MRSA SSTI infection than BALB/c with an increased skin lesion size and bacterial burden at the infection site. These findings are consistent with prior studies and indicated that a humanized mouse model is more suitable to study the MRSA infection in humans than BALB/c [31]. Human CD45 is a receptor for the PVL of SA, which indicates that cells, which express human CD45 possibly are more susceptible to MRSA infection [32]. We also observed that neutrophils from humanized mice were more susceptible to MRSA supernatant than that from BALB/c indicating humanized mice mimicing the human immune system, and is a more suitable animal model for studying MRSA SSTI. 

Using this humanized mouse as an in vivo model for MRSA SSTI, we found the *VraSR* is associated with the virulence of MRSA ST30 strain. The expression of *VraSR* was reported to be upregulated in response to phagocytosis of PMNs and inactivation of *VraSR* made the strain more susceptible to phagocytosis by PMNs indicating the role of *VraSR* in the response to human immune system [33]. In our humanized mouse model, when infected with the isogenic mutant strain ST30Δ*vraR,* a significant reduction in the skin lesion size was observed. This finding is in line with the prior study in *Streptococcus suis*, where the *VraSR* mutant was more susceptible to human PMNs, oxidant, and lysozyme than wildtype *S. suis*, and deletion of *VraSR* had greatly attenuated virulence in a mouse infection model [24]. Our study of CA-MRSA ST30 strain has showed reduction of virulence and increased susceptibility towards the human immune system in the ST30Δ*vraR* strain. 

The adhesion and internalization of the ST30Δ*vraR* mutant significantly decreased in the 3D-skin model and in the skin lesion of the humanized mouse model as compared to their respective parental wildtype strains. No significant difference in the release of cytokines was observed in the humanized mouse model, which is consistent of the prior study showing that VraSR had no influence on the production of many inflammatory cytokines at an early stage of *S. suis* infection [24]. However, in the 3D-skin model we observed a significant difference in the release of IL-6 after 24 h of infection. These findings indicate that the *VraSR* play a role in colonization. As expected, the TUNEL assay revealed Δ*vraR* induced significantly lower cell death when compared to the respective wildtype indicating the role *VraSR* during invasion of the skin tissue. The results of our study will cumulatively provide a foundation for further exploration of the molecular pathways that *VraSR* regulate in skin and soft tissue infection.

## Supplementary Materials

The following supporting information can be downloaded at: https://www.mdpi.com/article/10.3390/biomedicines10010035/s1, Figure S1: Generation of humanized mouse model. (A) Histogram of CD34+ cells isolated from human blood using CD34 microbeads. (B) Flow cytometry dot plots of blood cells used for the engraftment in humanized mice. Dot plot of proportion of human (Hu CD45) and mouse CD45 (Ms CD45) cells. Peripheral blood was collected from mice at 14 weeks post engraftment with human CD34+ HSCs and stained with antibodies to mouse (APC, red) and human CD45 (FITC, green). The level of engraftment equal to % CD45+ human cells divided by the sum of % CD45+ human cells plus % CD45+ mouse cells, Figure S2: Agarose Gel electrophoresis of colony PCR for ΔvraR gene knockout confirmation. (A) Lane 1 is the 100 bp ladder and lane 2 is the 200 bp amplicon for wildtype vraR. (B) Lane 1 is the 100 bp ladder and lane 2 is the 1100 bp amplicon for mutant vraR with intron insertion. (C) Lane 1 is the 100 bp ladder, lane 2 is the 550 bp PCR amplicon (positive control) using pNL9164 seq F and pNl9164 seq R primers as the positive control, and lane 3 showing the absence of the amplicon band confirming the curing of pNL9164 plasmid in knockout clone, Figure S3: Cytokine responses in humanized mice. Humanized mice were infected with 10^7^ CFU MRSA. (A) Human TNF-α, (B) IL-6, (C) IL-1β and (D) IL-8. Data are expressed as mean ± SD from one representative experiment. * *p* < 0.05; Table S1: List of primers used in the Construction of the isogenic mutant, Section S1: Supplemental Information.

## References

[1] Queck S.Y., Jameson-Lee M., Villaruz A.E., Bach T.H.L., Khan B.A., Sturdevant D.E., Ricklefs S.M., Li M., Otto M. (2008). RNAIII-Independent Target Gene Control by the agr Quorum-Sensing System: Insight into the Evolution of Virulence Regulation in Staphylococcus aureus. Mol. Cell.

[2] McCaig L.F., McDonald L.C., Mandal S., Jernigan D.B. (2006). Staphylococcus aureus-associated skin and soft tissue infections in ambulatory care. Emerg. Infect. Dis..

[3] Ho P.L., Chuang S.K., Choi Y.F., Lee R.A., Lit A.C.H., Ng T.K., Que T.L., Shek K.C., Tong H.K., Tse C.W.S. (2008). Community-associated methicillin-resistant and methicillin-sensitive Staphylococcus aureus: Skin and soft tissue infections in Hong Kong. Diagn. Microbiol. Infect. Dis..

[4] Talan D.A., Krishnadasan A., Gorwitz R.J., Fosheim G.E., Limbago B., Albrecht V., Moran G.J. (2011). Comparison of staphylococcus aureus from skin and soft-tissue infections in us emergency Department patients, 2004 and 2008. Clin. Infect. Dis..

[5] Matsuo M., Kato F., Oogai Y., Kawai T., Sugai M., Komatsuzawa H. (2010). Distinct two-component systems in methicillin-resistant Staphylococcus aureus can change the susceptibility to antimicrobial agents. J. Antimicrob. Chemother..

[6] Mascher T. (2006). Intramembrane-sensing histidine kinases: A new family of cell envelope stress sensors in Firmicutes bacteria. FEMS Microbiol. Lett..

[7] Boyle-Vavra S., Yin S., Jo D.S., Montgomery C.P., Daum R.S. (2013). VraT/YvqF is required for methicillin resistance and activation of the VraSR regulon in Staphylococcus aureus. Antimicrob. Agents Chemother..

[8] Otto M. (2014). Staphylococcus aureus toxins. Curr. Opin. Microbiol..

[9] Alonzo F., Torres V.J. (2013). Bacterial Survival Amidst an Immune Onslaught: The Contribution of the Staphylococcus aureus Leukotoxins. PLoS Pathog..

[10] Parker D. (2017). Humanized mouse models of Staphylococcus aureus infection. Front. Immunol..

[11] Spaulding A.R., Salgado-Pabón W., Kohler P.L., Horswill A.R., Leung D.Y.M., Schlievert P.M. (2013). Staphylococcal and streptococcal superantigen exotoxins. Clin. Microbiol. Rev..

[12] Yao J., Zhong J., Fang Y., Geisinger E., Novick R.P., Lambowitz A.M. (2006). Use of targetrons to disrupt essential and nonessential genes in Staphylococcus aureus reveals temperature sensitivity of Ll.LtrB group II intron splicing. RNA.

[13] Monk I.R., Shah I.M., Xu M., Tan M.W., Foster T.J. (2012). Transforming the untransformable: Application of direct transformation to manipulate genetically Staphylococcus aureus and Staphylococcus epidermidis. mBio.

[14] Gu H., Huang L., Wong Y.P., Burd A. (2010). HA modulation of epidermal morphogenesis in an organotypic keratinocyte-fibroblast co-culture model. Exp. Dermatol..

[15] Van Pham P., Le H.T., Vu B.T., Pham V.Q., Le P.M., Phan N.L.C., Van Trinh N., Nguyen H.T.L., Nguyen S.T., Nguyen T.L. (2016). Targeting breast cancer stem cells by dendritic cell vaccination in humanized mice with breast tumor: Preliminary results. Onco-Targets Ther..

[16] Tseng C.W., Biancotti J.C., Berg B.L., Gate D., Kolar S.L., Müller S., Rodriguez M.D., Rezai-Zadeh K., Fan X., Beenhouwer D.O. (2015). Increased Susceptibility of Humanized NSG Mice to Panton-Valentine Leukocidin and Staphylococcus aureus Skin Infection. PLoS Pathog..

[17] García C.A., Alcaraz E.S., Franco M.A., De Rossi B.N.P. (2015). Iron is a signal for Stenotrophomonas maltophilia biofilm formation, oxidative stress response, OMPs expression, and virulence. Front. Microbiol..

[18] Lee B.Y., Singh A., David M.Z., Bartsch S.M., Slayton R.B., Huang S.S., Zimmer S.M., Potter M.A., Macal C.M., Lauderdale D.S. (2013). The economic burden of community-associated methicillin-resistant Staphylococcus aureus (CA-MRSA). Clin. Microbiol. Infect..

[19] Yang S.J., Bayer A.S., Mishra N.N., Meehl M., Ledala N., Yeaman M.R., Xiong Y.Q., Cheung A.L. (2012). The Staphylococcus aureus two-component regulatory system, grars, senses and confers resistance to selected cationic antimicrobial peptides. Infect. Immun..

[20] Mainiero M., Goerke C., Geiger T., Gonser C., Herbert S., Wolz C. (2010). Differential target gene activation by the Staphylococcus aureus two-component system saeRS. J. Bacteriol..

[21] Boyle-Vavra S., Yin S., Daum R.S. (2006). The VraS/VraR two-component regulatory system required for oxacillin resistance in community-acquired methicillin-resistant Staphylococcus aureus. FEMS Microbiol. Lett..

[22] Fournier B., Klier A., Rapoport G. (2001). The two-component system ArlS-ArlR is a regulator of virulence gene expression in Staphylococcus aureus. Mol. Microbiol..

[23] O’Loughlin J.L., Spinner J.L., Minnich S.A., Kobayashi S.D. (2010). Yersinia pestis two-component gene regulatory systems promote survival in human neutrophils. Infect. Immun..

[24] Chang P., Li W., Shi G., Li H., Chen H., Bei W. (2018). The VraSR regulatory system contributes to virulence in Streptococcus suis via resistance to innate immune defenses. Virulence.

[25] Gao C., Dai Y., Chang W., Fang C., Wang Z., Ma X. (2019). VraSR has an important role in immune evasion of Staphylococcus aureus with low level vancomycin resistance. Microbes Infect..

[26] Dai Y., Chang W., Zhao C., Peng J., Xu L. (2017). VraR Binding to the Promoter Region of agr Inhibits Its Function in Vancomycin-Intermediate Staphylococcus aureus (VISA) and Heterogeneous VISA. Antimicrob. Agents Chemother..

[27] Kim H.K., Missiakas D., Schneewind O. (2014). Mouse models for infectious diseases caused by Staphylococcus aureus. J. Immunol. Methods.

[28] Salgado-Pabón W., Schlievert P.M. (2014). Models matter: The search for an effective Staphylococcus aureus vaccine. Nat. Rev. Microbiol..

[29] Shultz L.D., Brehm M.A., Victor Garcia-Martinez J., Greiner D.L. (2012). Humanized mice for immune system investigation: Progress, promise and challenges. Nat. Rev. Immunol..

[30] Knop J., Hanses F., Leist T., Archin N.M., Buchholz S., Gläsner J., Gessner A., Wege A.K. (2015). Staphylococcus aureus Infection in Humanized Mice: A New Model to Study Pathogenicity Associated with Human Immune Response. J. Infect. Dis..

[31] Prince A., Wang H., Kitur K., Parker D. (2017). Humanized mice exhibit increased susceptibility to staphylococcus aureus pneumonia. J. Infect. Dis..

[32] Tromp A.T., Van Gent M., Abrial P., Martin A., Jansen J.P., De Haas C.J.C., Van Kessel K.P.M., Bardoel B.W., Kruse E., Bourdonnay E. (2018). Human CD45 is an f-component-specific receptor for the staphylococcal toxin Panton-Valentine leukocidin. Nat. Microbiol..

[33] Voyich J.M., Braughton K.R., Sturdevant D.E., Whitney A.R., Saïd-Salim B., Porcella S.F., Long R.D., Dorward D.W., Gardner D.J., Kreiswirth B.N. (2005). Insights into Mechanisms Used by Staphylococcus aureus to Avoid Destruction by Human Neutrophils. J. Immunol..

