# Supplementary material for: VraSR Regulatory System Contributes to the Virulence of Community-Associated Methicillin-Resistant Staphylococcus aureus (CA-MRSA) in a 3D-Skin Model and Skin Infection of Humanized Mouse Model
Source: Biomedicines. 2021 Dec 24;10(1):35. doi: 10.3390/biomedicines10010035 (PMC8772825; doi:10.3390/biomedicines10010035)
Supplement: Supplementary file 1 [file biomedicines-10-00035-s001.zip › Supplemental Figures.pptx]

## Slide 1
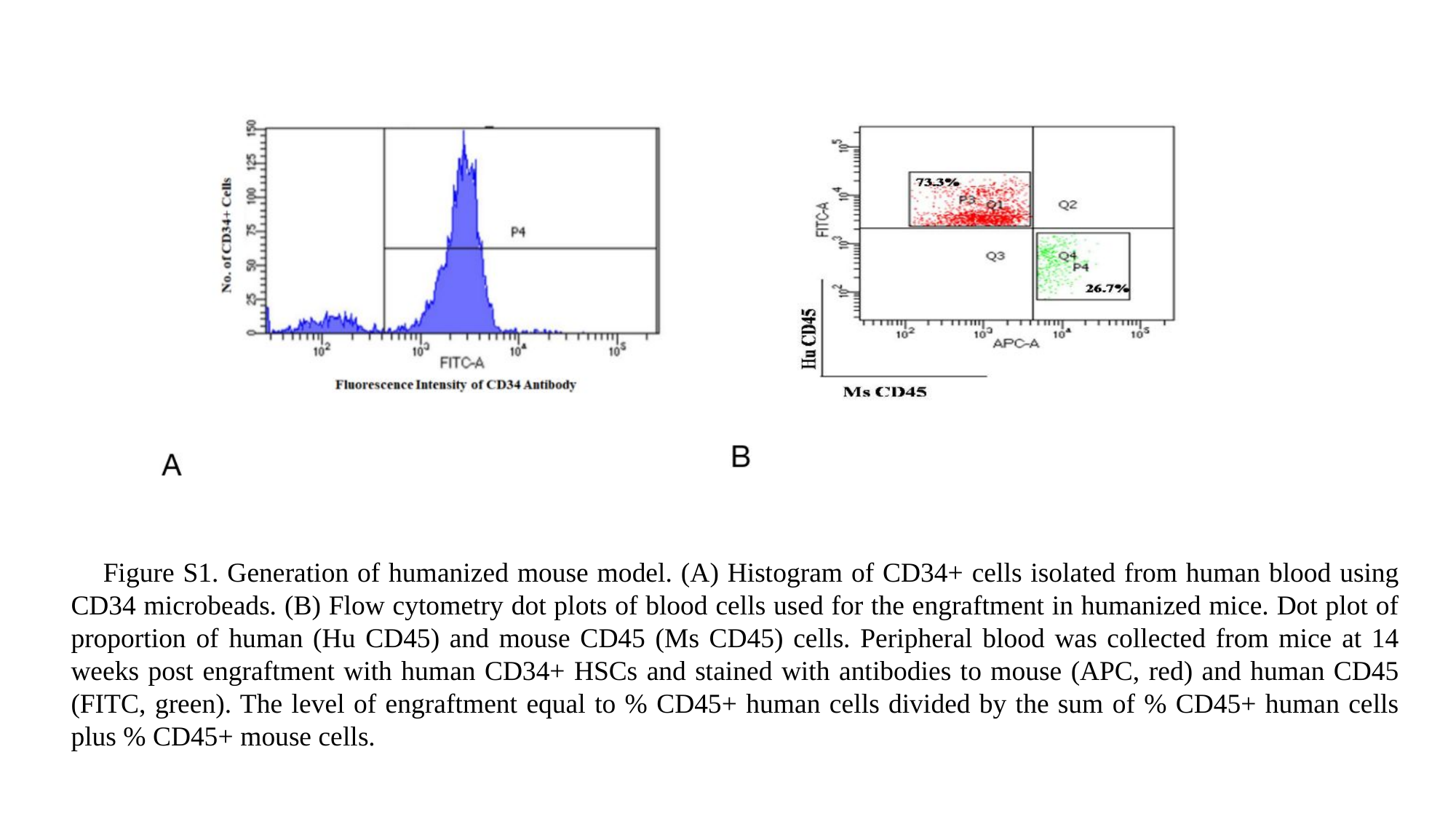

Figure S1. Generation of humanized mouse model. (A) Histogram of CD34+ cells isolated from human blood using CD34 microbeads. (B) Flow cytometry dot plots of blood cells used for the engraftment in humanized mice. Dot plot of proportion of human (Hu CD45) and mouse CD45 (Ms CD45) cells. Peripheral blood was collected from mice at 14 weeks post engraftment with human CD34+ HSCs and stained with antibodies to mouse (APC, red) and human CD45 (FITC, green). The level of engraftment equal to % CD45+ human cells divided by the sum of % CD45+ human cells plus % CD45+ mouse cells.

## Slide 2
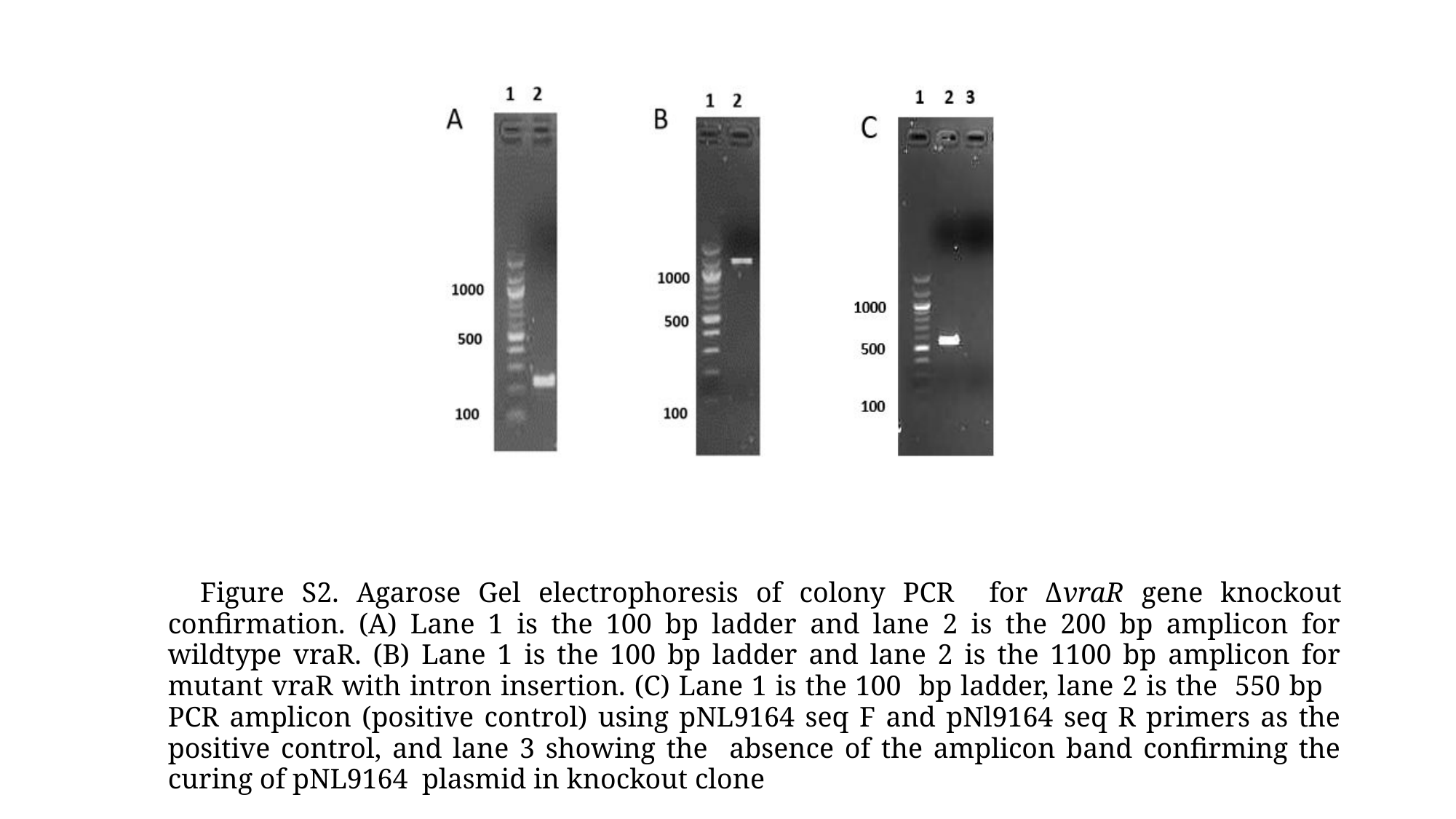

Figure S2. Agarose Gel electrophoresis of colony PCR for ΔvraR gene knockout confirmation. (A) Lane 1 is the 100 bp ladder and lane 2 is the 200 bp amplicon for wildtype vraR. (B) Lane 1 is the 100 bp ladder and lane 2 is the 1100 bp amplicon for mutant vraR with intron insertion. (C) Lane 1 is the 100 bp ladder, lane 2 is the 550 bp PCR amplicon (positive control) using pNL9164 seq F and pNl9164 seq R primers as the positive control, and lane 3 showing the absence of the amplicon band confirming the curing of pNL9164 plasmid in knockout clone

## Slide 3
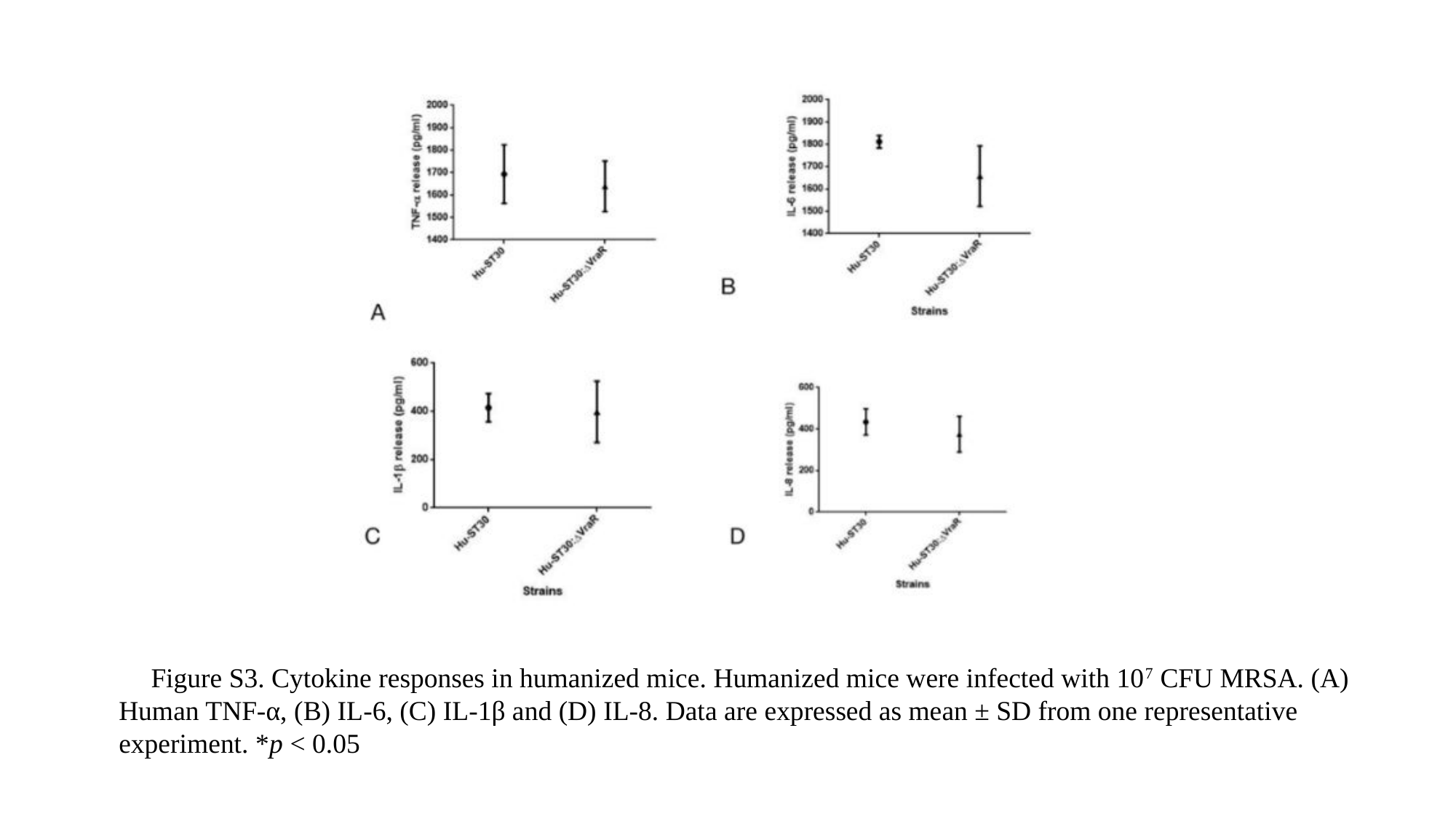

Figure S3. Cytokine responses in humanized mice. Humanized mice were infected with 107 CFU MRSA. (A) Human TNF-α, (B) IL-6, (C) IL-1β and (D) IL-8. Data are expressed as mean ± SD from one representative experiment. *p < 0.05
