# Supplementary material for: VraSR Regulatory System Contributes to the Virulence of Community-Associated Methicillin-Resistant Staphylococcus aureus (CA-MRSA) in a 3D-Skin Model and Skin Infection of Humanized Mouse Model
Source: Biomedicines. 2021 Dec 24;10(1):35. doi: 10.3390/biomedicines10010035 (PMC8772825; doi:10.3390/biomedicines10010035)
Supplement: Supplementary file 1 [file biomedicines-10-00035-s001.zip › Supplemental Table.pdf]

Supplemental Information

S1. KGM is composed of 1:3 mixture of Ham's F12 (Gibco) and DMEM (Gibco), 10% FBS(Gibco) , 5 ug/mL insulin (Sigma) , 1.8 x 10^{-4} M adenine sulfate (Sigma), 10^{-10} M cholera toxin (Sigma), 0.4 ug/mL hydrocortisone (Sigma), 1 ng/mL EGF (Gibco) and 0.1% BSA (Sigma).

Supplemental Table

Table S1. List of primers used in the Construction of the isogenic mutant

Name	Sequence
IBS	AAAAAAGCTTATAATTATCCTTAGGTTCCACTGTTGTGCGCCC AGATAGGGTG
EBS1d	CAGATTGTACAAATGTGGTGATAACAGATAAGTCACTGTTACT AACTTACCTTTCTTTGT
EBS2	TGAACGCAAGTTTCTAATTTCGATTGAACCTCGATAGAGGAA AGTGTCT
flank F	TTTTAAAATGTAACTATCGACACC
flank R	TGATCATGAAATGGTACGTATAG
pNL9164 seq F	AGAAAGAAGTGAAGGTCAATGTCTGAAC
pNl9164 seq R	GAGTACTCCGTACCCTTGCAAGATTT
